# Adaptive Value of Phenological Traits in Stressful Environments: Predictions Based on Seed Production and Laboratory Natural Selection

**DOI:** 10.1371/journal.pone.0032069

**Published:** 2012-03-05

**Authors:** Benjamin Brachi, Carla Aimé, Cédric Glorieux, Joel Cuguen, Fabrice Roux

**Affiliations:** Laboratoire Génétique et Evolution des Populations Végétales, UMR CNRS 8198, Université des Sciences et Technologies de Lille – Lille 1, Villeneuve d'Ascq, France; Ecole Normale Supérieure de Lyon, France

## Abstract

Phenological traits often show variation within and among natural populations of annual plants. Nevertheless, the adaptive value of post-anthesis traits is seldom tested. In this study, we estimated the adaptive values of pre- and post-anthesis traits in two stressful environments (water stress and interspecific competition), using the selfing annual species *Arabidopsis thaliana*. By estimating seed production and by performing laboratory natural selection (LNS), we assessed the strength and nature (directional, disruptive and stabilizing) of selection acting on phenological traits in *A. thaliana* under the two tested stress conditions, each with four intensities. Both the type of stress and its intensity affected the strength and nature of selection, as did genetic constraints among phenological traits. Under water stress, both experimental approaches demonstrated directional selection for a shorter life cycle, although bolting time imposes a genetic constraint on the length of the interval between bolting and anthesis. Under interspecific competition, results from the two experimental approaches showed discrepancies. Estimation of seed production predicted directional selection toward early pre-anthesis traits and long post-anthesis periods. In contrast, the LNS approach suggested neutrality for all phenological traits. This study opens questions on adaptation in complex natural environment where many selective pressures act simultaneously.

## Introduction

Local adaptation is the divergent evolution of phenotypic traits in populations (or demes in a metapopulation context) leading to values that maximize fitness in the local biotic and abiotic contexts [1]. Understanding patterns of local adaptation first requires identifying traits that are potentially important for fitness [2], [3]. Explaining the observed patterns of phenotypic diversity then requires identifying the ecological factors that exert selective pressures on those traits in the different populations or demes [4], [5], [6]. Validating both the adaptive values of phenotypic traits and the importance of ecological factors in the process of local adaptation requires experiments in controlled conditions that expressly test those factors [1]. In nature, the intensity of selective pressure varies both at the spatial scale among geographically close natural populations, and even among individuals within a population [7], [8] and at the temporal scale [9]. Variation in the intensity of selective pressure may have an effect on the strength and nature of selection, but also on the relative importance of selection on different traits [10], [11], [12], [13].

In annual plant species, abiotic and biotic factors define a favorable period during which individuals must complete their life cycle to maximize reproduction [14], [15], [16], [17], [18], [19]. The life cycle of annual plants is composed of two successive phases. The first phase corresponds to vegetative growth, during which plants accumulate resources. Then, individuals shift to the second phase of their life cycle, i.e., the reproductive phase [20]. This second phase starts with the opening of the first flower (i.e. anthesis) and ends when all the fruits are mature and the plant dies. The adaptive value of pre-anthesis traits, spanning the vegetative-growth phase, has been extensively studied. Germination timing can directly influence seedling survival and the phenotypic expression of post-germination traits [21], [22], [23]. Genetic variation in germination timing may account for up to 90% of the variation in fitness [24], [25]. Appropriate timing of reproduction relative to environmental cues is crucial to ensure that offspring are produced in good conditions [26], [27]. First, bolting time, which corresponds to the onset of elongation of the reproductive internodes of the leaf zone [20], is often correlated with latitude, suggesting a selective cline linked to globally varying environmental factors, such as photoperiod, temperature or precipitation [28], [29]. Second, the interval between bolting and anthesis has been shown to be adaptive to crowding [12], [13].

Post-anthesis traits can also be major components of fitness, but testing their adaptive values has received less attention. In outcrossing species, flowering, i.e., the time elapsed between the appearance of the first flower and the senescence of the last flower, is thought to be related to the number of mates [30]. Optimal flowering in natural populations may result from complex interactions with mutualists, such as pollinators, and antagonists, such as pollinator-transmitted diseases [31]. Natural variation for flowering has also been observed in selfing species suggesting that ecological factors, such as herbivory [32], pre-dispersal seed predation [31], [33], or seasonal variation in the likelihood of seed dispersion [23], may be selective agents that act on flowering. Often referred to as seed-fill duration in crop species [34], [35], [36], the duration of the reproductive period, i.e., the time elapsed from anthesis to the maturity of all fruits, is related to the number and quality of seeds [36], [37], [38], [39]. Seed number is often used as a proxy for female fitness and seed quality is known to influence seedling establishment [33], [40].

The goal of this study was to estimate the adaptive values of seven pre-anthesis and post-anthesis traits under two types of environmental stress (biotic and abiotic) with four levels of intensity, using a segregating progeny of *Arabidopsis thaliana*. *A. thaliana* is a mostly selfing annual plant with a worldwide distribution [41]. This ubiquitous species encounters a great variety of ecological conditions [42], [43], [44] and displays extensive natural variation in pre-anthesis and post-anthesis traits [13], [45]. The two types of environmental stress considered in this study, i.e., water stress and interspecific competition, are major determinants of the favorable period for completing the life cycle in annual plants [23]. To estimate the adaptive values of seven phenological traits in two stressful environments with different levels of intensity, we used two complementary approaches: (1) we measured the relationship between phenological traits and a major fitness component in a selfing annual plant species, i.e., seed production; and (2) we conducted experimental evolution (LNS, i.e. Laboratory natural selection) [46]. Measuring a major fitness component helps predict the strength and nature of selection on phenological traits [33], [47], [48]. As total fitness may not be correctly approximated through a set of measurable traits [49], experimental evolution provides a means to monitor the micro-evolutionary dynamics of phenological traits in artificial populations without using a proxy for fitness [1]. We asked the following questions: (1) Is there any evidence for adaptation in phenological traits, especially in post-anthesis traits? (2) Do the strength and nature of selection depend on the environmental stress tested and its intensity? (3) Are results from the two approaches (i.e., direct measurement of a major fitness component and experimental evolution) consistent?

## Results

### Predicting the effects of natural selection: measuring a major component of fitness

To estimate the adaptive values of seven pre-anthesis and post-anthesis traits under two types of environmental stress (biotic and abiotic) with different levels of intensity, we first measured the relationship between phenological traits and seed production. We used a set of 160 Recombinant Inbred Lines (RILs) produced from a cross between two natural accessions, i.e., Col-0 and Ri-0. RILs resulted from two generations of intercrosses, followed by 5–6 generations of single seed descent, making the 160 RILs quasi-homozygous genotypic lines. This Ri-0×Col-0 RIL family was expected to broaden the range of phenological combinations on which natural selection may act (see Materials and Methods section). We set up an experiment involving 2,460 plants to grow the set of 160 RILs in five different treatments: ‘control’ treatment; ‘water stress’ treatment with two intensities chosen to simulate severe and moderate drought, named hereafter ‘severe water stress’ and ‘moderate water stress’, respectively; ‘competition’ treatment with two intensities of interspecific competition with the annual bluegrass *Poa annua*, named hereafter ‘moderate competition’ and ‘intense competition’, respectively. *P. annua* is frequently associated with *A. thaliana* in natural plant communities in France (database on weed communities, http://www2.dijon.inra.fr/bga/umrbga/). The 160 RILs were phenotyped for seed production (FITNESS) and seven phenological traits spanning the life cycle of *A. thaliana*: germination timing (GERM), bolting time (BT), time to anthesis (ANT), interval between bolting and anthesis (INT), flowering (FLO), reproductive period duration (RP), flowering-to-reproductive period ratio (FRR = FLO/RP). FRR may indicate a trade-off between seed number and seed quality [38], [39]. Low FRR would indicate that seed quality is favored, while high FRR would indicate that seed number is favored.

#### Treatment and genotype effects on phenological traits and fitness

**Figure 1:**
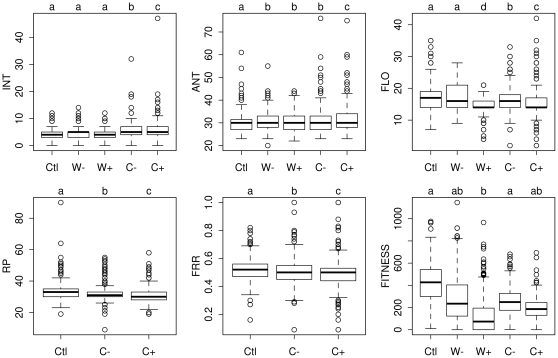
Effect of water stress and competition on phenological traits and fitness. INT: interval between bolting and anthesis, ANT: anthesis, FLO: flowering, RP: reproductive period duration, FRR: flowering-to-reproductive period ratio, FITNESS: total silique length as a proxy of seed production. INT, FT, FP and RP are expressed in days. FITNESS is expressed in millimeters. Ctl: ‘control’ treatment. W−: ‘moderate water stress’ treatment. W+: ‘severe water stress’ treatment. C−: ‘moderate competition’ treatment. C+: ‘intense competition’ treatment. For each phenotypic trait, different letters indicate different phenotypic means among treatments after a Tukey's test of multiple comparisons of means (*P* = 0.05). Data are not available for RP and FRR in the two ‘water stress’ treatments (see Material and Methods).

A ‘genotype’ effect was highly significant for all phenological traits and for fitness (Table S1 and S2). A ‘treatment’ effect was not significant for germination timing and bolting time but significant for the interval between bolting and anthesis, anthesis, flowering, reproductive period duration and flowering-to-reproductive period ratio, as well as on seed production (Table S1). Compared to the ‘control’ treatment, plants under stress flowered slightly later, had a shorter flowering duration, a shorter reproductive period, a lower flowering-to-reproductive period ratio, and produced fewer seeds (especially under severe water stress; Figure 1). More stressful conditions (i.e., severe water stress and intense competition) had greater effects on the decrease of flowering, reproductive period duration and flowering-to-reproductive period ratio means than moderately stressful conditions (i.e., moderate water stress and moderate competition). The interval between bolting and anthesis appeared to increase slightly in the ‘competition’ treatments. No ‘treatment×genotype’ interaction was detected, suggesting the absence of genetic variation in the reaction norms of phenological traits and fitness across the five treatments.

#### Selection estimates

Because environmental covariances between phenotypic traits and fitness may bias selection estimates [50], both phenotypic and genotypic selection analyses were performed to directly measure natural selection on phenological traits. We calculated standardized selection differentials (S) for each phenological trait within each of the five experimental treatments. Since selection differentials include both direct selection on a trait and indirect selection due to selection acting on correlated traits, we performed a multivariate selection gradient analysis to distinguish direct from indirect selection on individual phenological traits. Linear partial regression coefficients (β) for each phenological trait indicate the strength and trend of directional selection, while quadratic regression coefficients (γ) for each phenological trait estimate stabilizing (i.e., negative coefficient) or disruptive (i.e., positive coefficient) selection.

For both phenotypic and genotypic selection analyses, standardized selection differentials (S) and linear partial regression coefficients (β) always had the same sign when both parameters were statistically significant (Table 1 and Table 2). Since β represents the relative contribution of an individual trait on fitness accounting for indirect selection on other phenological traits [48], only the results based on multivariate selection gradient analysis (β and γ) are presented.

**Table 1:** Phenotypic selection analysis with selection differentials (S), selection gradients (β), quadratic selection coefficients (γ) for phenological traits in each treatment.

Treatment	Trait	S (SE)	β (SE)	γ (SE)
Control	GERM	−0.12 (0.02)***	−0.11 (0.02)***	−0.01 (0.02)
	BT	−0.13 (0.02)***	−0.17 (0.02)***	0.01 (0.01)
	INT	−0.05 (0.02)	−0.09 (0.02)***	−0.05 (0.02)*
	ANT	−0.16 (0.02)***	NE	NE
	FLO	0.01 (0.02)	−1.53 (0.41)***	0.26 (0.07)***
	RP	0.03 (0.02)	1.07 (0.30)***	−0.09 (0.04)*
	FRR	0.01 (0.02)	1.00 (0.26)***	−0.17 (0.04)***
Moderate water stress	GERM	−0.10 (0.04)**	**−0.19 (0.03)** ***	0.01 (0.02)
	BT	−0.53 (0.03)***	**−0.64 (0.03)** ***	**0.13 (0.04)** **
	INT	−0.08 (0.04)	**−0.26 (0.03)** ***	0.02 (0.03)
	ANT	−0.57 (0.03)***	NE	NE
	FLO	0.20 (0.04)***	**−0.1 (0.03)** ***	−0.06 (0.04)
Severe water stress	GERM	−0.08 (0.05)	**−0.36 (0.04)** ***	−0.05 (0.02)
	BT	−0.73 (0.04)***	**−1.35 (0.05)** ***	**0.34 (0.03)** ***
	INT	−0.21 (0.05)***	**−0.57 (0.04)** ***	−0.01 (0.04)
	ANT	−0.84 (0.04)***	NE	NE
	FLO	0.25 (0.05)***	**−0.46 (0.04)** ***	0.07 (0.02)
Moderate competition	GERM	−0.16 (0.02)***	−0.15 (0.02)***	−0.01 (0.01)
	BT	−0.19 (0.02)***	**−0.26 (0.02)** ***	**0.08 (0.03)** **
	INT	−0.14 (0.02)***	−0.12 (0.02)***	**0.02 (0.01)**
	ANT	−0.23 (0.02)	NE	NE
	FLO	0.07 (0.02)**	**0.35 (0.29)**	**−0.08 (0.06)**
	RP	0.06 (0.02)**	**−0.18 (0.18)**	**0.02 (0.03)**
	FRR	0.04 (0.02)	**−0.19 (0.21)**	**0.02 (0.04)**
Intense competition	GERM	−0.14 (0.02)***	−0.11 (0.03)***	−0.00 (0.02)
	BT	−0.2 (0.03)***	−0.24 (0.03)***	**0.10 (0.04)** *
	INT	−0.2 (0.03)***	**−0.19 (0.04)** ***	0.02 (0.04)
	ANT	−0.24 (0.03)***	NE	NE
	FLO	0.03 (0.02)	**0.74 (0.32)** *	**−0.15 (0.06)** *
	RP	0.06 (0.02)	**−0.31 (0.16)**	**0.02 (0.02)**
	FRR	0 (0.02)	**0.54 (0.25)** *	**0.04 (0.04)**

GERM: germination timing, BT: bolting time, INT: interval between bolting and anthesis, ANT: anthesis, FLO: flowering, RP: reproductive period duration, FRR: flowering-to-reproductive period ratio. Selection is stabilizing when γ<0 and disruptive when γ>0. Standard errors (SE) are in parentheses. Values in bold indicate significantly different selection coefficients compared to the ‘control’ treatment. Because ANT integrates BT and INT, ANT was not included in polynomial regressions.

* 0.05>*P*>0.01,

** 0.01>*P*>0.001,

*** *P*<0.001. NE: not estimated.

**Table 2:** Genotypic selection analysis with selection differentials (S), selection gradients (β), quadratic selection coefficients (γ) for phenological traits in each treatment.

Treatment	Trait	S (SE)	β (SE)	γ (SE)
Control	GERM	−0.13 (0.02)***	−0.10 (0.02)***	−0.06 (0.02)*
	BT	−0.09 (0.02)***	−0.09 (0.03)***	0 (0.02)
	INT	−0.06 (0.02)	−0.07 (0.02)**	−0.06 (0.02)*
	ANT	−0.12 (0.02)***	NE	NE
	FLO	−0.01 (0.02)	−0.40 (0.25)	0.10 (0.08)
	RP	0.01 (0.03)	0.26 (0.18)	−0.01 (0.04)
	FRR	−0.02 (0.03)	0.27 (0.16)	−0.12 (0.06)*
Moderate water stress	GERM	−0.03 (0.04)**	−0.08 (0.04)*	**0.06 (0.02)** *
	BT	−0.35 (0.03)***	**−0.40 (0.03)** ***	**0.12 (0.04)** **
	INT	−0.02 (0.04)	−0.12 (0.03)***	0.02 (0.04)
	ANT	−0.38 (0.03)***	NE	NE
	FLO	−0.24 (0.04)***	**−0.14 (0.03)** ***	−0.08 (0.06)
Severe water stress	GERM	−0.04 (0.07)	−0.19 (0.05)***	−0.02 (0.02)
	BT	−0.56 (0.05)***	**−0.93 (0.05)** ***	**0.30 (0.06)** ***
	INT	−0.18 (0.07)**	**−0.36 (0.04)** ***	−0.02 (0.04)
	FT	−0.64 (0.04)***	NE	NE
	FLO	0.17 (0.07)**	**−0.30 (0.05)** ***	0.08 (0.06)
Moderate competition	GERM	−0.12 (0.02)***	−0.12 (0.02)***	0 (0.02)
	BT	−0.14 (0.02)***	**−0.19 (0.02)** ***	**0.12 (0.04)** **
	INT	−0.11 (0.02)***	−0.11 (0.03)***	**0.06 (0.02)** **
	ANT	−0.17 (0.02)***	NE	NE
	FLO	0.02 (0.03)	−0.31 (0.20)	0.10 (0.04)*
	RP	0.01 (0.03)	0.26 (0.15)	−0.04 (0.02)
	FRR	0.02 (0.03)	0.25 (0.14)	−0.14 (0.04)***
Intense competition	GERM	−0.08 (0.03)**	−0.08 (0.03)*	0.02 (0.02)
	BT	−0.11 (0.03)***	−0.16 (0.03)***	0.08 (0.02)*
	INT	−0.09 (0.03)**	−0.15 (0.04)***	**0.04 (0.02)** *
	ANT	−0.13 (0.02)***	NE	NE
	FLO	−0.04 (0.03)	0.02 (0.10)	−0.04 (0.04)
	RP	0 (0.03)	0.01 (0.06)	0 (0.04)
	FRR	−0.05 (0.03)	−0.02 (0.08)	−0.02 (0.04)

GERM: germination timing, BT: bolting time, INT: interval between bolting and anthesis, ANT: anthesis, FLO: flowering, RP: reproductive period duration, FRR: flowering-to-reproductive period ratio. Selection was stabilizing when γ<0 and disruptive when γ>0. Standard errors (SE) are in parentheses. Values in bold indicate significantly different selection coefficients compared to the ‘control’ treatment. Because ANT integrates BT and INT, ANT was not included in polynomial regressions.

* 0.05>*P*>0.01,

** 0.01>*P*>0.001,

*** *P*<0.001. NE: not estimated.

**Figure 2:**
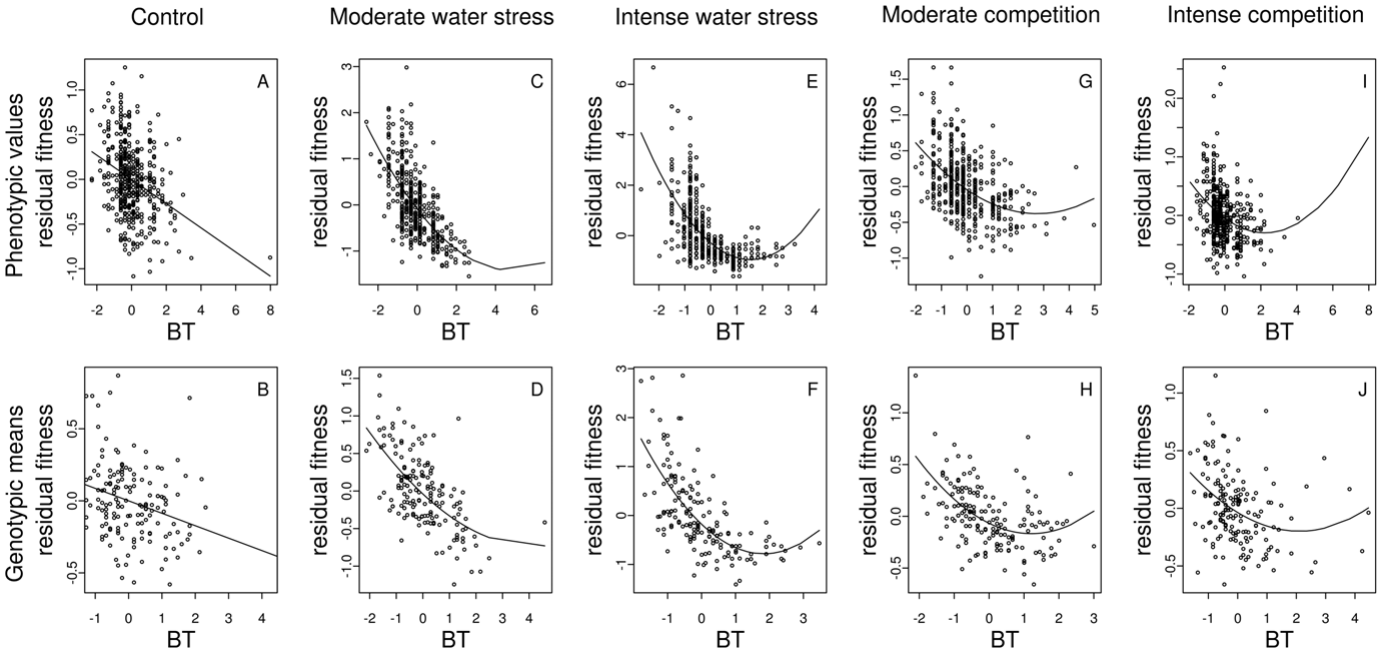
Relationship between bolting time and fitness for each treatment at both phenotypic and genotypic levels. For illustration purposes, a polynomial regression including both linear and quadratic terms described either in Table 1 (phenotypic level) or in Table 2 (genotypic level) was first performed including all traits but bolting time. Then, a second polynomial regression including the linear and quadratic terms associated with bolting time was run on the residual fitness of the first polynomial regression. The black lines were drawn using the parameters from this second polynomial regression. BT: bolting time. BT is expressed in standardized values.

**Figure 3:**
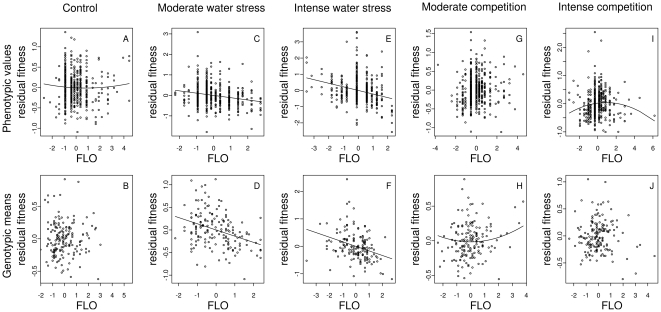
Relationship between flowering and fitness for each treatment at phenotypic and genotypic levels. For illustration purposes, a polynomial regression including both linear and quadratic terms described either in Table 1 (phenotypic level) or in Table 2 (genotypic level) was first performed including all traits but flowering duration. Then, a second polynomial regression including the linear and quadratic terms associated with flowering duration was run on the residual fitness of the first polynomial regression. The black lines were drawn using the parameters from this second polynomial regression. FLO: flowering. FLO is expressed in standardized values. The absence of a black line indicates that there is no significant linear or quadratic relationship between fitness and flowering (see Tables 1 and 2).

At the phenotypic level, selection was detected for each trait in at least one of the five treatments. The total variance in fitness among individuals was best explained by phenological variation in the ‘water stress’ treatments compared to the other treatments (*R* ^2^ from polynomial regression; Control: *R* ^2^_pheno_ = 0.25, Moderate water stress: *R* ^2^_pheno_ = 0.54, Severe water stress: *R* ^2^_pheno_ = 0.65, Moderate competition: *R* ^2^_pheno_ = 0.37, Intense competition: *R* ^2^_pheno_ = 0.25). In the ‘control’ treatment (Table 1), slight directional selection was detected, favoring earlier phenotypes for germination timing, bolting time (Figure 2A) and the interval between bolting and anthesis. Directional selection was detected with trends for shorter flowering (Figure 3A), a longer reproductive period and a higher flowering-to-reproductive period ratio, suggesting selection for higher seed number.

Different patterns of directional selection were observed between the two types of environmental stress. In the ‘water stress’ treatments (Table 1), the coefficients of directional selection toward early phenotypes for bolting time (Figures 2C and 2E) and the interval between bolting and anthesis were greater than the coefficient of directional selection for a shorter flowering (Figures 3C and 3E). For all phenological traits, coefficients of directional selection in the ‘water stress’ treatments were significantly different from the coefficients of directional selection estimated in the ‘control’ treatment (Table 1, Figures 2 and 3). For all phenological traits, the intensity of directional selection increased significantly with the intensity of water stress (Figures 2 and 3, Table S3). In the ‘competition’ treatment (Table 1), directional selection favored earlier phenotypes for germination timing, bolting time (Figures 2G and 2I) and the interval between bolting and anthesis. For post-anthesis traits (flowering, reproductive period duration and flowering-to-reproductive period ratio), no directional selection was observed under moderate competition (Table 1, Figure 3G). Selection changed direction in the ‘intense competition’ treatment compared to the ‘control’ treatment for two post-anthesis traits (flowering and flowering-to-reproductive period ratio). Selection favored longer flowering under intense competition in comparison to the control treatment, i.e. intermediate flowering duration (Figures 3A and 3I) and higher flowering-to-reproductive period ratio. While significant differences for selection intensity were detected between each ‘competition’ treatment and the ‘control’ treatment, no differences were detected between the two ‘competition’ treatments (Table S3).

In the ‘control’ treatment, stabilizing selection was detected for the interval between bolting and anthesis, reproductive period duration and flowering-to-reproductive period ratio, while disruptive selection was detected for flowering duration (Table 1, Figure 3A). Similarly to directional selection, different patterns of stabilizing and disruptive selection were observed in the two types of environmental stress. In contrast with patterns observed in the ‘control’ treatment, significant disruptive selection was detected for bolting time under both water stress and competition treatments (Figures 2C, 2E, 2G and 2I). The intensity of disruptive selection for bolting time significantly increased with the intensity of water stress but not with the intensity of competition (Table S3). In contrast to the ‘control’ treatment, stabilizing selection was detected for flowering under the ‘intense competition’ treatment (Figure 3I).

At the genotypic level, percentages of fitness variation explained by phenological variation were almost identical to the percentages estimated at the phenotypic level (*R* ^2^ from polynomial regression; Control: *R* ^2^_geno_ = 0.26, Moderate water stress: *R* ^2^_geno_ = 0.54, Severe water stress: *R* ^2^_geno_ = 0.65, Moderate competition: *R* ^2^_geno_ = 0.37, Intense competition: *R* ^2^_geno_ = 0.25). For the pre-anthesis traits, directional and stabilizing/disruptive selection coefficients based on genotypic means (Table 2) were generally consistent with results obtained for raw phenotypic data under the five treatments (Table 1). Directional selection favoring earlier phenotypes was detected for germination timing, bolting time (Figures 2B, 2D, 2F, 2H and 2J) and the interval between bolting and anthesis. Compared to the phenotypic selection analysis, significant disruptive selection was predicted for the interval between bolting and anthesis in both ‘moderate’ and ‘intense’ competition treatments (Table 2). This disruptive selection had the opposite sign and significantly differed from patterns observed in the ‘control’ treatment (i.e., significant stabilizing selection).

With the exception of flowering, which showed patterns of selection comparable to what was predicted from the phenotypic selection analysis in the ‘water stress’ treatments (Tables 1 and 2, Figures 3C and 3D, Figures 3E and 3F), differences between phenotypic and genotypic selection analyses were detected for post-anthesis traits. Under the ‘control’ treatment, no directional selection was predicted for post-anthesis traits (Figure 3B). Quadratic selection coefficients for those traits were also non-significant when looking at genotypic means. Only flowering-to-reproductive period ratio appeared to show weak, but still significant, stabilizing selection (Table 2). In the two ‘competition’ treatments, no directional selection was predicted for post-anthesis traits (Table 2). In contrast to the phenotypic selection analysis, disruptive and stabilizing selections were detected for flowering (Figure 3H) and flowering-to-reproductive period ratio under moderate competition, respectively (Table 2).

As in the phenotypic selection analysis, directional selection predicted from genotypic means was significantly stronger for germination timing, bolting time (Figures 2D and 2F), the interval between bolting and anthesis and flowering (Figures 3D and 3F) in the ‘intense water stress’ treatment than in the ‘moderate water stress’ treatment (Table S4). No difference in selection strength was identified between the two ‘competition’ treatments.

#### Trait heritabilities

In the ‘control’ treatment, significant heritabilities were detected for all traits (except germination timing), with the three most heritable traits being bolting time, anthesis and seed production (FITNESS; Table 3). These traits also had the strongest heritabilities in the four treatments, whereas post-anthesis traits and the interval between bolting and anthesis generally had lower heritability values (Table 3).

**Table 3:** Estimates of broad-sense heritability (*H^2^*) for seven phenological traits in *Arabidopsis thaliana* in five treatments.

		Water stress		Competition	
Trait	Control	Moderate	Severe	Moderate	Intense
GERM	0.20^ns^	0.38***	0.25*	0.33^ns^	0.10**
BT	0.51***	0.38***	0.44***	0.57***	0.40***
INT	0.26*	0.17^ns^	0.03^ns^	0.12*	0.20^ns^
ANT	0.55***	0.47***	0.46***	0.60***	0.45***
FLO	0.03*	0.42***	0.30**	0.25^ns^	0.25*
RP	0.14**	NE	NE	0.22^ns^	0.29*
FRR	0.31*	NE	NE	0.09^ns^	0.21^ns^
FITNESS	0.47***	0.42***	0.52***	0.28***	0.46**

GERM: germination timing, BT: bolting time, INT: interval between bolting and anthesis, ANT: anthesis, FLO: flowering, RP: reproductive period duration, FRR: flowering-to-reproductive period ratio, FITNESS: total silique length as a proxy of seed production. Asterisks indicate a significant RIL effect in the analyses of variance performed to estimate *H^2^*.

* 0.05>*P*>0.01,

** 0.01>*P*>0.001,

*** *P*<0.001,

ns : non-significant. NE: not estimated.

#### Phenotypic and genetic correlations among phenological traits

For each treatment, pairwise phenotypic correlations were similar to pairwise genetic correlations (Table 4). Flowering was strongly associated (>0.6) with both reproductive period duration and flowering-to-reproductive period ratio; reproductive period duration and flowering-to-reproductive period ratio were not significantly correlated to each other. The pre-anthesis traits bolting time and the interval between bolting and anthesis were less correlated to the post-anthesis traits flowering and reproductive period duration in the ‘control’, ‘moderate competition’ (except bolting time with flowering) and ‘intense competition’ treatments, suggesting some level of independence between the vegetative phase and the reproductive phase in these treatments.

**Table 4:** Correlations among phenological traits in five treatments.

Treatment	Trait	GERM	BT	INT	FLO	RP	FRR
**Control**	GERM		−0.04	0.06	−0.15**	−0.08	−0.13**
	BT	0.04		−0.27***	0.12	−0.07	0.26***
	INT	0.06	−0.28***		0.11	0.15	0.03
	FLO	−0.20*	0.11*	0.06		0.65***	0.75***
	RP	−0.15	−0.04	0.10*	0.63***		0.04
	FRR	−0.14	0.24***	0	0.75***	0.03	
**Moderate competition**	GERM		−0.07	0.20***	−0.27***	−0.17***	−0.23***
	BT	−0.13		0.15	0.37***	0.18*	0.37***
	INT	0.17*	0.17***		0.05	0.08	0.07
	FLO	−0.26**	0.28***	−0.11*		0.68***	0.67***
	RP	−0.08	−0.02	−0.15**	0.69***		−0.05
	FRR	−0.26***	0.37***	0.09*	0.72***	0.03	
**Intense competition**	GERM		0.18***	0.07	−0.09	−0.08	−0.06
	BT	0.15		0.13	0.10	−0.09	0.20*
	INT	0.05	−0.01		−0.09	−0.07	−0.06
	FLO	0.02	0.13**	−0.10*		0.66***	0.82***
	RP	0.06	−0.06	−0.11*	0.58***		0.22*
	FRR	−0.01	0.21***	−0.08	0.84***	0.09	
**Moderate water stress**	GERM		−0.01	−0.01	−0.13***		
	BT	0.07		−0.23***	0.22*		
	INT	−0.05	−0.26***		−0.09		
	FLO	−0.08	0.23***	−0.09			
**Severe water stress**	GERM		−0.13***	0.10*	−0.23***		
	BT	−0.08		−0.20*	−0.57***		
	INT	0.06	−0.26***		−0.07		
	FLO	−0.24***	−0.54***	−0.15***			

Within each treatment, phenotypic and genetic Pearson correlations are given above and below the diagonal, respectively. GERM: germination timing, BT: bolting time, INT: interval between bolting and anthesis, FLO: flowering, RP: reproductive period duration, FRR: flowering-to-reproductive period ratio. The correlations between RP or FRR and the other phenological traits were not computed for the two water stress treatments (see Material and Methods section).

* 0.05>*P*>0.01,

** 0.01>*P*>0.001,

*** *P*<0.001. NA: not available.

Treatment was found to induce shifts in phenological associations. The sign of genetic correlation between bolting time and flowering shifted across treatments. It was positive in the ‘moderate competition’ and ‘moderate water stress’ treatments, non-significant in the ‘control’ and ‘intense competition’ treatments, and negative in the ‘intense water stress’ treatment, suggesting a trade-off between bolting time and flowering in the latter treatment. The sign of genetic correlations between bolting time and the interval between bolting and anthesis also shifted across treatments. The genetic correlation between bolting time and the interval between bolting and anthesis was positive in the ‘moderate competition’ treatment, non-significant in the ‘intense competition’ treatment, and negative in the ‘control’ and both ‘water stress’ treatments, suggestion a trade-off between bolting time and the interval between bolting and anthesis in these latter treatments.

#### Genetic constraints

Combining genotypic selection analysis (Table 2) and study of pairwise genetic correlations (Table 4) forecasted genetic constraints on phenological evolution that differed among treatments. In the ‘control’ and both ‘water stress’ treatments, selection for earlier bolting and a shorter interval between bolting and anthesis was predicted from selection analysis, although bolting time and the interval between bolting and anthesis were negatively correlated. This suggests that the optimal phenotype might not be available within the phenotypic variation of the Ri-0×Col-0 RIL family. Similarly, in the ‘severe water stress’ treatment, selection for earlier germination, earlier bolting and a shorter flowering was predicted by selection analysis although flowering was negatively correlated with both germination timing and bolting time.

### Laboratory natural selection for monitoring phenotypic evolution across generations

In a second approach to estimate the adaptive values of seven phenological traits in two stressful environments with different levels of intensity, we conducted a Laboratory Natural Selection (LNS) experiment [46] of four discrete non-overlapping generations. The LNS experiment involved nine treatments and ten experimental populations for each treatment (Figure S1). In the first generation G_0_, each experimental population was composed of the 160 genetic lines of the Ri-0×Col-0 RIL family. The nine treatments correspond to a ‘control’ treatment (i.e. no stress); a ‘water stress’ treatment with four intensities of water stress expected to simulate mild to severe drought, named hereafter W1 to W4, respectively; a ‘competition’ treatment with four intensities of interspecific competition with the annual bluegrass *P. annua* expected to simulate moderate to intense competition, named hereafter C1 to C4, respectively. *A. thaliana* and *P. annua* seeds were scattered in the LNS experiment, while a homogeneous interspecific competition was simulated in the approach measuring a major component of fitness (see Material and Methods section).

To study the micro-evolutionary dynamics of phenological traits over four experimental generations, five out of the ten populations for which we have kept seeds were randomly chosen for each treatment, totaling 45 experimental populations per generation (Figure S1). The same five populations were followed over the four generations (Figure S1). A set of 1,710 plants were grown for each type of environmental stress (i.e., water stress and competition). For a given environmental stress, each set of 1,710 plants included experimental populations from the four intensities of stress (n = 20) and experimental populations from the control treatment (n = 5). Within each set of 1,710 plants, each ‘experimental population×generation’ combination was represented by 12 individuals. All plants were phenotyped for germination timing, bolting time, the interval between bolting and anthesis, time to anthesis, flowering, reproductive period duration and flowering-to-reproductive period ratio. Hereafter, for each type of environmental stress, treatment intensity corresponds to the four intensities of stress and the control treatment (i.e. no stress).

No significant ‘population (treatment intensity)’ or ‘population (treatment intensity)×generation’ effect was detected in the ‘water stress’ treatment (Table 5), suggesting parallel micro-evolutionary dynamics among experimental populations within each treatment intensity. In the ‘competition’ environment, the ‘population (treatment intensity)’ and ‘population (treatment intensity)×generation’ effects were significant for bolting time, suggesting that drift cannot be excluded as a cause for evolution of bolting time in the experimental populations (Table 5).

**Table 5:** Evolution of phenological traits in experimental evolution populations of *Arabidopsis thaliana* in the ‘water stress’ and ‘competition’ treatments.

Treatment type	Source	GERM	BT	INT	FLO	RP	FRR
Water stress	Block	**0.0001**	0.396	**0.0003**	0.059	0.216	0.209
	Generation	**0.0001**	**0.0005**	0.993	**0.0001**	**0.0001**	0.109
	treatment intensity	**0.0001**	**0.0001**	0.069	0.078	0.552	**0.009**
	generation×treatment intensity	0.091	**0.004**	0.691	0.780	0.956	0.799
	population (treatment intensity)	0.290	0.639	0.906	0.811	0.74	0.95
	population (treatment intensity)×generation	0.963	0.129	0.836	0.445	0.436	0.51
Competition	block	0.860	0.712	0.588	**0.003**	0.095	**0.009**
	generation	**0.0001**	**0.0005**	**0.008**	**0.004**	**0.017**	0.063
	treatment intensity	0.571	0.339	0.484	0.982	0.967	0.994
	generation×treatment intensity	0.555	0.673	0.96	0.965	0.83	0.954
	population (treatment intensity)	0.071	**0.018**	0.44	0.375	0.141	0.734
	population (treatment intensity)×generation	0.11	**0.003**	0.875	0.285	0.872	0.333

For each environmental stress treatment, values indicate 95^th^ percentile of *P*-values obtained for each tested factor after simulating the initial composition of the theoretical G_0_ populations 500 times (see Material and Methods section). Values in bold indicate significant *P*-values. GERM: germination timing, BT: bolting time, INT: interval between bolting and anthesis, FLO: flowering, RP: reproductive period duration, FRR: flowering-to-reproductive period ratio.

No ‘generation’ effect was found for the interval between bolting and anthesis and flowering-to-reproductive period ratio in the ‘water stress’ treatment (Table 5, Figure S2), while a significant decrease along generations was observed for germination timing, bolting time, flowering and reproductive period duration (Table 5, Figure S2). A ‘generation’ effect was found significant for germination timing, bolting time, the interval between bolting and anthesis, flowering and reproductive period duration in the ‘competition’ environment (Table 5). However, the change in phenotypic mean from one generation to the next appeared to be stochastic, following no particular trend (see, e.g., bolting time, the interval between bolting and anthesis and reproductive period duration in Figure S2).

**Figure 4:**
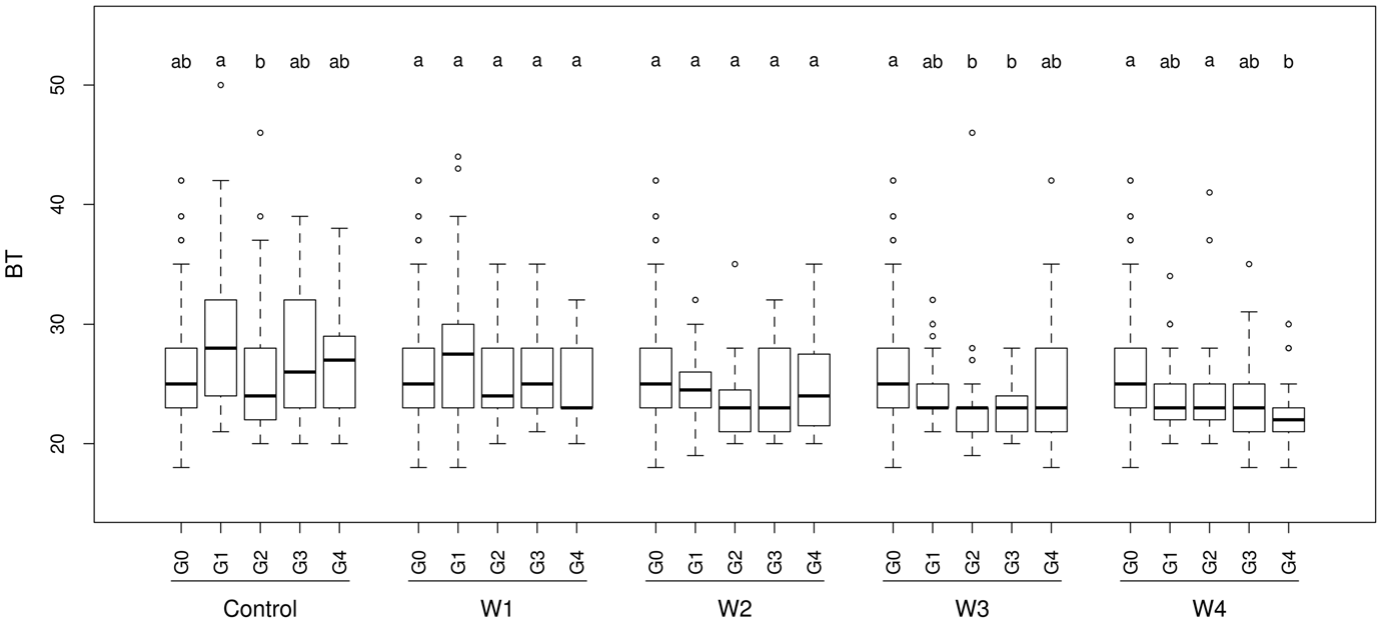
Evolution of bolting time in experimental populations of *Arabidopsis thaliana* in the ‘water stress’ treatments. Control: ‘control’ treatment. W1–W4 correspond to the four water stress intensities simulating mild to severe drought (i.e. bottom-watering stopped either 46, 39, 32 or 25 days after sowing for W1, W2, W3 and W4, respectively). BT (bolting time) is expressed in days. G0: initial experimental generation, G1–G4: four successive experimental generations. For each treatment, different letters indicate different phenotypic means among generations after a Tukey's test of multiple comparisons of means (*P* = 0.05). For each treatment, a box-plot representing raw data is given for each experimental generation.

‘Treatment intensity’ had a significant effect on germination timing, bolting time and flowering-to-reproductive period ratio in the ‘water stress’ treatment (Table 5). Compared to the ‘control’ treatment, lower flowering-to-reproductive period ratios were observed in all four intensities of water stress. Selection for early germination and early bolting increased with the intensity of water stress (Figure 4). The interaction ‘treatment intensity×generation’ was also found to have a significant effect on bolting time (Table 5), suggesting different micro-evolutionary dynamics of bolting time across treatment intensities (Figure 4). Selection for early bolting appeared significantly more efficient in the two most severe water stress treatments (Figure 4). In the ‘competition’ environment, no significant effect was found for either the ‘treatment intensity’ or the ‘treatment intensity×generation’ factors (Table 5).

## Discussion

### Adaptive value of phenological traits in *A. thaliana*

Estimation of seed production, a proxy for fitness, in the different environmental stress conditions tested in this study predicted evolution toward a shorter vegetative phase. In stressful environments, such as those characterized by limited water, light or mineral resources, annual plants are expected to evolve toward a reduction in the length of their life cycle to maximize their fitness [17], [18], [23], [51]. Early germination has been found to be favored because biological space can be occupied faster and thereby constitute a competitive advantage over later germinants [21], [23]. Earlier bolting or anthesis has also been shown to constitute a strategy for escaping drought and avoiding competition [52], herbivory [32] or pre-dispersal seed predators [31], [33].

To a lesser extent, disruptive selection for bolting time was also detected in both water stress and interspecific competition suggesting that late bolting might constitute another phenological optimum in both environmental stresses. Late bolting in *A. thaliana* has been demonstrated to be associated with higher water use efficiency (WUE) which may lead to a strategy of dehydration avoidance in presence of drought stress [53]. The functional alleles of the *Flowering Locus C* (*FLC*) gene which caused later flowering have been proposed to have pleiotropic effects on WUE by increasing carbon stable isotope ratio δ^13^C [53]. Interestingly, both parental lines of the RIL family used in this study have a functional allele at *FLC* [54] which may confer an advantage to late bolting RILs in our water stress conditions. Late bolting plants are also known to bolt at a larger size with more leaves [55], which may confer an advantage to individuals in competition with neighboring plants [23], [56].

In our study, selection for shorter pre-anthesis periods and earlier reproduction was also detected in the ‘control’ treatment, probably due to the high density of *A. thaliana* individuals and intraspecific competition. However, this interpretation contrasts with a previous finding whereby intraspecific competition simulated under greenhouse conditions appeared to favor late-bolting individuals [12]. However, the biological material in the Dorn *et al.* [12] study was based on 36 accessions coming from four natural populations and, in contrast to our study, no crosses were done. Strong genetic correlation among traits in these accessions may have led to extensive indirect selection, i.e. selection acts on a trait correlated to the focal one [48]. The RIL family used in this study may have a wider range of phenotypes with fewer and/or lower genetic correlations among traits, allowing selection to act on a larger space of potential phenotypes [57].

In contrast to pre-anthesis traits, the adaptive value of post-anthesis traits has received less empirical attention [30], [31], [37], [58]. Differences in selection on post-anthesis traits in *A. thaliana* were detected between the two environmental stresses simulated in this study. For example, an intermediate flowering appeared to be advantageous in intense interspecific competition, whereas evolution toward a shorter flowering was predicted in the environment simulating different drought intensities. This difference can be attributed to different strategies of resource allocation. Under our water stress conditions, discontinuing bottom-watering may have hastened the timing of mortality. This sudden lack of water may have favored genotypes for which all the resources accumulated during the vegetative phase are reallocated to seed production in a short post-anthesis period.

Since inflorescences may contribute more than rosettes to lifetime carbon gain in *A. thaliana* [55], natural selection may favor genotypes with long flowering. This might be especially relevant in a competitive environment where shade may limit photosynthesis at the rosette stage. On the other hand, as previously suggested in *A. thaliana*, a prolonged competition for resources may lead to a reduction of the environmental quality which, in turn, may counteract the positive effect of a long flowering [13]. Interestingly, in complex natural environments, many selective pressures probably act simultaneously on the same trait. Selection may therefore remain undetected because antagonistic selective pressures may slow down the evolution of a given trait.

Although rarely tested, different intensities of selective pressure appeared to influence the strength and/or nature of selective processes but not the relative importance of selection on phenological traits scored in this study. More intense selective pressures appeared to lead to stronger selection (directional and/or quadratic), certainly as a consequence of the reduction of light and/or nutrients availability. Differences in initial pathogen density [59], various levels of crowding [12], or seasonality of water stress [18] have already been shown to differentially affect selection on anthesis. Adaptive values of phenological traits should therefore be studied as reaction norms of selection under a gradient of selective pressure.

### Lessons from laboratory natural selection

Laboratory natural selection (LNS) is thought to complement approaches that directly measure a fitness component [46]. Through the LNS approach, selection can be detected and its dynamics can be studied over several generations without using a proxy for fitness. Coupling this experimental evolution approach with measurements of a fitness component, the mechanistic bases of selection can be better understood, because both direct and indirect components of selection on individual traits can be detected [48]. However, in either approach, the main target of selection may be an unmeasured trait correlated with the traits of interest, such as plant size with the length of flowering [60].

Compared to a direct measure of seed production, the LNS approach in this study has nonetheless one caveat which concerns environmental maternal effects. Although greenhouse conditions were regulated, temperature and light conditions may have varied slightly from one experimental generation to another. In plant species, maternal environments are well documented to mainly affect early rather than late stages of plant development, [61]. This is particularly relevant in *A. thaliana* where environmental maternal effects are known to influence seed quality [62], [63], which in turn may influence germination date [64], [65] and vegetative growth [66]. If environmental maternal effects exist throughout our experiment and affect phenological traits, they may influence the pattern of trait evolution across the four generations. For example, if offspring phenotypes resulting from environmental maternal effects fit to the local phenotypic optimum, the genetic response to selection may be well overestimated in our LNS approach. Although we cannot disentangle the relative importance of genetic *vs.* maternal effects on the phenological evolution in our experimental populations, it should be however noted that environmental maternal effects were found not to persist for more than one generation in *A. thaliana* [62]. Despite this caveat, the LNS experiment under water stress validated most of the results predicted by estimation of seed production in the two ‘water stress’ treatments. Early germination, early bolting and a short flowering were selected for in the experimental populations. The absence of selection on the interval between bolting and flowering was also predicted by a genetic constraint between this trait and bolting time. Since selection coefficients were higher for bolting time than for the interval between bolting and flowering, natural selection may have first operated on bolting time.

In the ‘competition’ treatment, results from LNS and estimation of seed production were less consistent. While estimation of seed production suggested directional selection on bolting time in all ‘competition’ treatments, LNS suggested neutrality for this phenological trait. Significant differences among experimental populations within a particular ‘competition’ treatment suggested a potential genetic drift effect. This interpretation is further supported by the observation of stochastic evolution for bolting time (and the other phenological traits) across generations. Stochastic evolution was also associated with high within-population variance that did not seem to evolve over generations (data not shown). Three hypotheses can be posited to explain the absence of phenological evolution in the competition environment. First, although significantly heritable, fitness variation was only weakly explained by the phenological traits scored in this study. This suggests that unmeasured traits might have greater adaptive value in competition. Seedling survival, relative growth rate and plant height, for example, are known to influence fitness when plants compete for nutrients and/or light [13], [23]. Second, traits with low heritability, such as flowering, appeared to contribute the most to fitness, resetting the initial range of phenological variation at each generation. Third, local differences in plant density may have occurred within each experimental population. In contrast to the approach estimating seed production, *A. thaliana* and *P. annua* seeds were sown at random in the LNS experiment. Spatial heterogeneity of selective pressures within populations may have enhanced the occurrence of non-adapted individuals in micro-refuges, therefore impeding the process of local adaptation [1].

Overall, we demonstrated that phenological traits including post-anthesis traits in *A. thaliana* can experience strong directional selection and, to a lesser extent, stabilizing or disruptive selection. These traits may therefore be potentially adaptive in natural populations. Selection varied in strength and nature according to the ecological factor tested and its intensity, suggesting that local adaptation of phenological traits is a highly complex phenomenon. Testing the combined effect of different selective pressures on the evolution of phenological traits certainly deserves further empirical investigations.

## Materials and Methods

### Plant material

We used a set of recombinant inbred lines (RILs) produced from a cross between two natural accessions, i.e., Col-0 (Versailles accession number: 186 AV) and Ri-0 (Versailles accession number: 160 AV). Col-0 is a common laboratory accession whose natural origin is uncertain [45], while Ri-0 comes from Richmond (Canada). The ecological characteristics of the habitats from which Col-0 and Ri-0 originate are unknown. RILs result from two generations of intercrosses, followed by six generations of single-seed descent (expected residual heterozygosity ∼0.78%). We chose the Ri-0×Col-0 RIL family from a set of 13 RIL families [67] for two reasons. Firstly, in a preliminary experiment, the Ri-0×Col-0 family showed the lowest values of genetic correlations among phenological traits (i.e., Pearson correlation coefficient (anthesis **-** flowering) = 0.2; Pearson correlation coefficient (anthesis **-** reproductive period duration) = 0.2; B. Brachi and F. Roux, unpublished results). Secondly, QTL mapping analyses indicated independent genetic bases for anthesis and flowering or reproductive period duration in the Ri-0×Col-0 RIL family under greenhouse conditions (B. Brachi and F. Roux, unpublished results). Both weak genetic correlations among phenological traits and different QTL patterns are expected to broaden the range of phenological combinations on which natural selection may act. The Ri-0×Col-0 RIL family is composed of 286 RILs. In this study, we used a set of 160 RILs corresponding to a core collection that maximized genetic diversity and recombination observed within the Ri-0×Col-0 RIL family [67]. To reduce maternal effects, the seeds were produced in the same controlled environment [67]. Further details on the creation of the Ri-0×Col-0 RIL family are available at the following website: http://dbsgap.versailles.inra.fr/vnat/.

### Predicting the effects of natural selection: measuring a major component of fitness

#### Greenhouse experiment A

To directly measure natural selection on phenological traits, an experiment involving 2,460 plants was set up according to a completely randomized design involving five treatments and three experimental blocks for each treatment. Each block was represented by a flat (28 cm×28 cm) filled with 1.5 kg of damp, standard culture soil (Huminsubstrat N3, Neuhaus). Each flat was an independent randomization of one replicate for each of the 160 RILs and two replicates per parental accession on a 13×13 grid of plants, positioned 2.5 cm apart. In each flat, the remaining five positions, corresponding to the four corners of the flat and one random position, were left empty. The density of plants was 2,100 plants/m^2^, a value similar to natural densities that seeds may experience when they are dispersed far from the maternal plant [68]. *A. thaliana* seeds that had not germinated 7 days after sowing were replaced by extra seedlings. Plants were grown at 20°C and under natural light supplemented by artificial light to provide a 16 hr photoperiod. During the whole growing period, flats were rotated every day to minimize potential effects of uneven lighting across the growth room.

In the ‘control’ treatment, plants were bottom-watered — without supplemental nutrients — as necessary until all individuals had senesced. The remaining four treatments involved two types of environmental stress, each with two intensities. These environmental stresses differed from the control treatment by a single environmental factor. In the ‘water stress’ treatment, all conditions were the same as for controls, except that bottom-watering was stopped either 32 days or 39 days after sowing (i.e., ∼7 and 14 days after the onset of flowering in a flat). These two water stress intensities were chosen to simulate severe (i.e., 32 days) and moderate (i.e., 39 days) drought, named hereafter ‘severe water stress’ and ‘moderate water stress’, respectively. In the ‘competition’ treatment, all conditions were the same as for controls, except that two intensities of interspecific competition with the annual bluegrass *Poa annua* were simulated. The two intensities of interspecific competition corresponded to densities of 2,270 and 4,540 *P. annua* plants/m^2^, named hereafter ‘moderate competition’ and ‘intense competition’, respectively. Each *A. thaliana* plant was surrounded by four and eight *P. annua* plants in the ‘moderate competition’ and ‘intense competition’ treatments, respectively. *P. annua* seeds were planted the same day as *A. thaliana* seeds. *P. annua* seeds that had not germinated 6 days after sowing were replaced by extra seedlings.

#### Measuring phenological traits and fitness

Seven phenological traits were measured during the experiment. Germination was monitored daily from when seeds were sown to 7 days after sowing. Germination date (GERM) was scored as the number of days from sowing to the opening of both cotyledons. Plants were monitored every 2 to 3 days for the remaining six phenological traits. Bolting time (BT) was scored as the number of days between germination and the date the inflorescence differentiated from leaves at a size <5 mm. Anthesis (ANT) was scored as the number of days between germination and the appearance of the first open flower. The interval between bolting and anthesis (INT) was measured as the difference between the bolting and anthesis dates. Flowering (FLO) was scored as the number of days between the appearance of the first flower and the senescence of the last flower on the main stem. The reproductive period duration (RP) was scored as the number of days between the appearance of the first flower and the maturation of the last fruit on the main stem. The flowering-to-reproductive period ratio (FRR) was calculated as the ratio between flowering (FLO) and reproductive period duration (RP).

Plant fitness was measured as total fruit length (FITNESS), which has been shown to be an accurate indicator of lifetime fitness for a selfing annual like *A. thaliana* because the length of a fruit (i.e., silique) strongly correlates with the number of seeds contained within it [69]. FITNESS was measured by counting the number of siliques produced on the primary shoot, the basal branches, and the primary branches on the primary shoot, and then multiplying these counts by an estimate of their corresponding silique length (calculated as the average of three representative siliques).

Since many plants did not complete their life cycle in the ‘moderate water stress’ and ‘severe water stress’ treatments, reproductive period duration and flowering-to-reproductive period ratio data are not available for these plants. Therefore, reproductive period duration and flowering-to-reproductive period ratio were only considered for statistical analyses in the ‘competition’ treatments. FITNESS estimates for plants growing in the ‘moderate water stress’ and ‘severe water stress’ treatments were only based on mature siliques.

#### Statistical analyses

For testing the effect of RIL genotype and treatment on the seven phenological traits and fitness, we used the following general linear model (GLM):
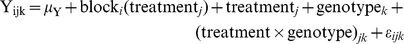
(1)


In this model, ‘Y’ is either one of the seven phenological traits or seed production (FITNESS), ‘μ’ is the overall mean, ‘block(treatment)’ accounts for differences in micro-environment among the three experimental blocks within each treatment, ‘treatment’ corresponds to the ‘control’ treatment and the two stress treatments (each with two stress intensities), ‘genotype’ measures the effect of the RIL genetic background, ‘treatment×genotype’ accounts for genetic differences in reaction norms among RILs, and ‘ε’ is the residual term. All factors were treated as fixed effects because the levels of each factor were not random samples from a population to which we intended to extrapolate. Raw data were Box-Cox transformed to satisfy the normality and equal variance assumptions of linear regression. Model fitting was conducted using the ‘nlme’ function implemented in the *R* environment (package nlme) [70]. The significance of the effects was tested after model selection based on a difference of two points in Akaike's information criterion (AIC) [71].

To test for environment-dependent natural selection on phenological traits, phenotypic [48] and genotypic selection analyses [50], [72] were performed. RIL means calculated within treatments were used for genotypic selection analyses. Standardized selection differentials (S) were obtained for all seven phenological traits within each treatment using simple regression:
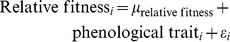
(2)


Where ‘Relative fitness’ is the relative fitness within each treatment calculated as the fitness estimate divided by the mean fitness estimate within that treatment, ‘μ’ is the constant, ‘phenological trait’ corresponds to phenological traits standardized within each treatment to have a mean of zero and a standard deviation of one, and ‘ε’ is the residual term. We corrected for multiple tests within treatments using a sequential Bonferroni criterion with k = 7, corresponding to the seven phenological traits.

Based on relative fitness and standardized phenological data, linear and quadratic selection differentials were calculated within each of the five experimental treatments using polynomial regressions. To correctly estimate stabilizing/disruptive selection gradients, quadratic regression coefficients were doubled [73]. All phenological traits were included in these analyses except anthesis, which integrates both bolting time and the interval between bolting and anthesis. Differences in linear and quadratic selection differentials between pairs of treatments were tested using analyses of covariance (ANCOVA). Significant ‘phenological trait×treatment’ and ‘(phenological trait)^2^×treatment’ interactions indicate varying directional and stabilizing/disruptive selection between treatments, respectively.

Using the formula adapted from [74], broad-sense heritability (*H^2^*) was estimated for each phenological trait in each of the five experimental treatments from the mean square (MS) obtained with the following general linear model (GLM):
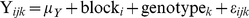
(3)


In this model, ‘Y’ is either one of the seven phenological traits or seed production (FITNESS), ‘μ’ is the overall mean, ‘block’ accounts for differences in the micro-environment among the three experimental blocks, ‘genotype’ measures the effect of the RIL genetic background, and ‘ε’ is the residual term.

Pairwise phenotypic and genotypic Pearson correlations among the seven phenological traits were calculated within each of the five experimental treatments. RIL means calculated within treatments were used for estimating genotypic correlations.

### Dynamics of selection: laboratory natural selection

#### Greenhouse experiment B

A Laboratory Natural Selection (LNS) experiment [46] of four generations was set up with nine treatments and ten experimental populations for each treatment (Figure S1). Each experimental population was represented by a flat (28 cm×28 cm) having high edges, ensuring that all flats were separated from each other. Each flat was filled with 1.5 kg of damp, standard culture soil (Huminsubstrat N3, Neuhaus). To initiate the initial generation (G_0_), each flat contained one replicate of each RIL, i.e., 160 seeds. Seeds were scattered at a mean plant density of 2,040 plants/m^2^. Over four generations (G_1_–G_4_) from October 2008 to November 2009, the populations were allowed to freely self-fertilize. At the end of each generation, seeds were carefully harvested and stored separately for each population (i.e., flat). To initiate the next generation, 160 seeds were randomly chosen and scattered in a new flat of equal size, the remaining seeds being stored at 4°C to preserve seed viability [65]. Throughout the experiment, generations were discrete, non-overlapping, and kept as isolated gene pools. Each generation was grown at 20°C under natural light supplemented with artificial light to provide a 16 hr photoperiod. In each generation, the 90 experimental populations were randomized over three greenhouse benches.

In the ‘control’ treatment, plants were bottom-watered without supplemental nutrients as necessary until all individuals had senesced. The remaining eight treatments involved two types of environmental stress, each with four intensities. Each of these environmental stresses differed from the ‘control’ treatment by a single environmental factor. In the ‘water stress’ environment, conditions were the same as for the ‘control’ treatment, except that bottom-watering was stopped either 25, 32, 39 or 46 days after sowing (i.e., ∼0, 7, 14 and 21 days after the onset of flowering in a flat on the first generation). These four intensities of water stress were expected to simulate mild (i.e., 46 days) to severe (i.e., 25 days) drought, named hereafter W1 to W4, respectively. In the ‘competition’ environment, conditions were the same as for the ‘control’ treatment, except that four intensities of interspecific competition with the annual bluegrass *P. annua* were simulated. The four intensities of interspecific competition corresponded to densities of 2,040, 4,080, 6,120 and 8,160 *P. annua* plants/m^2^, named hereafter C1 to C4, respectively. For each generation, *P. annua* seeds were scattered the same day as *A. thaliana* seeds.

Among all the populations from which we have kept seeds, we then randomly selected five populations for each treatment (Figure S1). The same 45 experimental populations (5 populations×9 treatments) were kept for the four generations of experimental evolution. A set of 1,710 plants was then grown for each type of environmental stress treatment (i.e., water stress and competition) according to a complete randomized design involving three blocks. Each block included nine arrays of 66 individual wells (Ø4 cm, vol. ∼38 cm^3^; TEKU, JP 3050/66) filled with damp standard culture soil (Huminsubstrat N3, Neuhaus). Each block was an independent randomization of (1) four seeds per experimental population (n = 25 or 5 populations×(4 stress intensities+1 control)) and per generation (n = 4, G_1_–G_4_), i.e., 4 seeds×25 populations×4 generations; (2) one replicate per RIL (n = 160) representing the initial generation G_0_; and (3) five replicates for each of the two parental accessions (Col-0 and Ri-0). In each block, the remaining 24 positions were left empty. Each ‘experimental population×generation’ combination was thus represented by 12 individuals (3 blocks×4 seeds/population/generation). *A. thaliana* seeds that had not germinated 11 days after sowing were replaced by extra seedlings. Plants were grown at 20°C under natural light supplemented with artificial light to provide a 16 hr photoperiod. Unlike the four generations of experimental evolution, all plants were watered until all individuals had senesced. During the whole growing period, arrays were rotated every day to minimize potential effects of uneven lighting across the growth room.

Seven phenological traits were measured for each individual. Germination timing (GERM) was monitored daily from the day seeds were sown to seven days after sowing. Plants were monitored every two or three days for the remaining six phenological traits, i.e., bolting time (BT), the interval between bolting and anthesis (INT), time to anthesis (ANT), flowering (FLO), reproductive period duration (RP) and flowering-to-reproductive period ratio (FRR).

#### Statistical analyses

For testing the effect of selection on the seven phenological traits, we used the following general linear models (GLMs) for each type of environmental stress (i.e., water stress and competition):
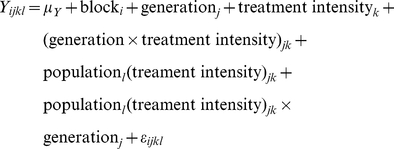
(4)


In this model, ‘Y’ is one of the seven phenological traits, ‘μ’ is the overall mean, ‘block’ accounts for differences in the micro-environment among the experimental blocks, ‘generation’ measures the rate of evolution of phenotypic mean across generations (G_0_–G_4_; see below for the creation of theoretical G_0_ populations, Figure S2), ‘treatment intensity’ corresponds to the difference among the five treatment intensities (no stress (i.e. 1 ‘control’ treatment)+4 stress intensities), ‘generation×treatment intensity’ accounts for differences in the rate of evolution of phenotypic mean among the five treatment intensities, ‘population (treatment intensity)’ measures the phenotypic reproducibility among replicates in a given treatment intensity, ‘population (treatment intensity)×generation’ measures the reproducibility of phenotypic evolution rate among replicates in a given treatment intensity, and ‘ε’ is the residual term. All factors were treated as fixed effects, except ‘population’ which was treated as a random effect. For calculating *F*-values, terms were tested over their appropriate denominators. Raw data were Box-Cox transformed to satisfy the normality and equal variance assumptions of linear regression. Model fitting was conducted using the PROC GLM procedure in SAS 9.1 (SAS Institute Inc., Cary, North Carolina, USA).

#### Theoretical populations for the initial generation G_0_

Because the initial generation G_0_ was represented by the 160 RILs with only one replicate per RIL and per block, theoretical G_0_ populations of 12 individuals were created to test for a ‘generation’ effect. For each type of environmental stress (i.e., ‘water stress’ and ‘competition’), the creation of theoretical G_0_ populations followed three steps (Figure S3). First, for each block, 25 sub-populations of four RILs were created with the condition that an individual should not be sampled twice. Second, 25 theoretical G_0_ populations of 12 individuals each were obtained by concatenating sub-populations among the three blocks with the condition that a given RIL cannot be present twice within the same population. This condition ensures that raw data were independent. Third, the 25 theoretical G_0_ populations were then randomly partitioned among the five treatments, i.e., control treatment and four stress intensities. Theoretical populations were created under the *R* environment [70].

To ensure that the initial composition of the theoretical G_0_ populations would not affect the output of the statistical analyses, this three-step process was repeated 500 times for each type of environmental stress. Analysis of variance according to equation (4) was run on each repeat, leading to a distribution of *P*-values for each tested factor. A factor was declared as significant when 95% of the *P*-values fell under the 0.05 *P*-value threshold.

### Data archiving

Any materials and information described in this manuscript have been deposited to the Dryad data repository: http://dx.doi.org/10.5061/dryad.mb0cd1bs

## Supporting Information

Figure S1 **Experimental design of Laboratory Natural Selection experiment.** W1 to W4: the four intensities of water stress expected to simulate mild (i.e., watering stopped 46 days after sowing) to severe (i.e., watering stopped 25 days after sowing) drought. C1 to C4: the four intensities of interspecific competition corresponding to densities of 2,040, 4,080, 6,120 and 8,160 *P. annua* plants/m^2^.
(TIF)

Figure S2 **Evolution of phenological traits in experimental populations of** ***Arabidopsis thaliana*** **in the ‘water stress’ and ‘competition’ treatments.** BT: bolting time, INT: interval between bolting and anthesis, RP: reproductive period duration. BT, INT and RP are expressed in days. G0: initial experimental generation, G1–G4: four successive experimental generations. For both treatments, raw data from the five intensities were pooled and are presented for each experimental generation.
(TIFF)

Figure S3 **Theoretical populations for the initial generation G_0_ of the experiment set up for each type of environmental stress treatment (i.e., water stress and competition).**
(TIF)

Table S1 **Effects of treatment and genotype on phenological traits and fitness.**
(DOC)

Table S2 **Model selection based on the Akaike's information criterion (AIC).**
(DOC)

Table S3 **Phenotypic selection analysis: comparison of selection gradients (β) and quadratic selection coefficients (γ) for phenological traits between the two intensities in each stress treatment (‘water stress’ and ‘competition’).**
(DOC)

Table S4 **Genotypic selection analysis: comparison of selection gradients (β) and quadratic selection coefficients (γ) for phenological traits between the two intensities in each stress treatment (‘water stress’ and ‘competition’).**
(DOC)
