# Supplementary figures and images for: Adaptive Value of Phenological Traits in Stressful Environments: Predictions Based on Seed Production and Laboratory Natural Selection
Source: PLoS One. 2012 Mar 5;7(3):e32069. doi: 10.1371/journal.pone.0032069 (PMC3293886; doi:10.1371/journal.pone.0032069)

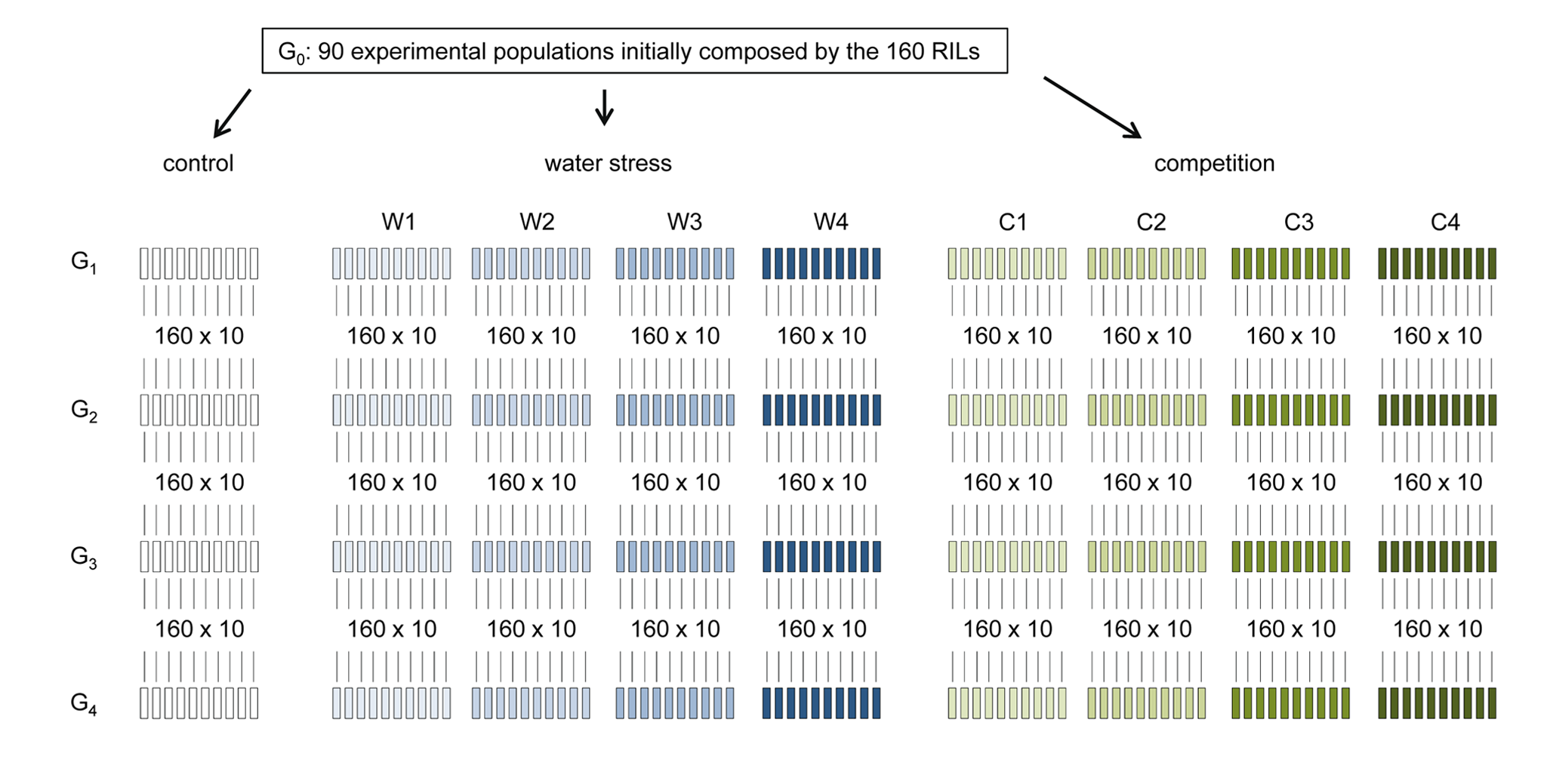

Supplement: Figure S1 — Experimental design of Laboratory Natural Selection experiment. W1 to W4: the four intensities of water stress expected to simulate mild (i.e., watering stopped 46 days after sowing) to severe (i.e., watering stopped 25 days after sowing) drought. C1 to C4: the four intensities of interspecific competition corresponding to densities of 2,040, 4,080, 6,120 and 8,160 P. annua plants/m2. (TIF) [file pone.0032069.s001.tif]

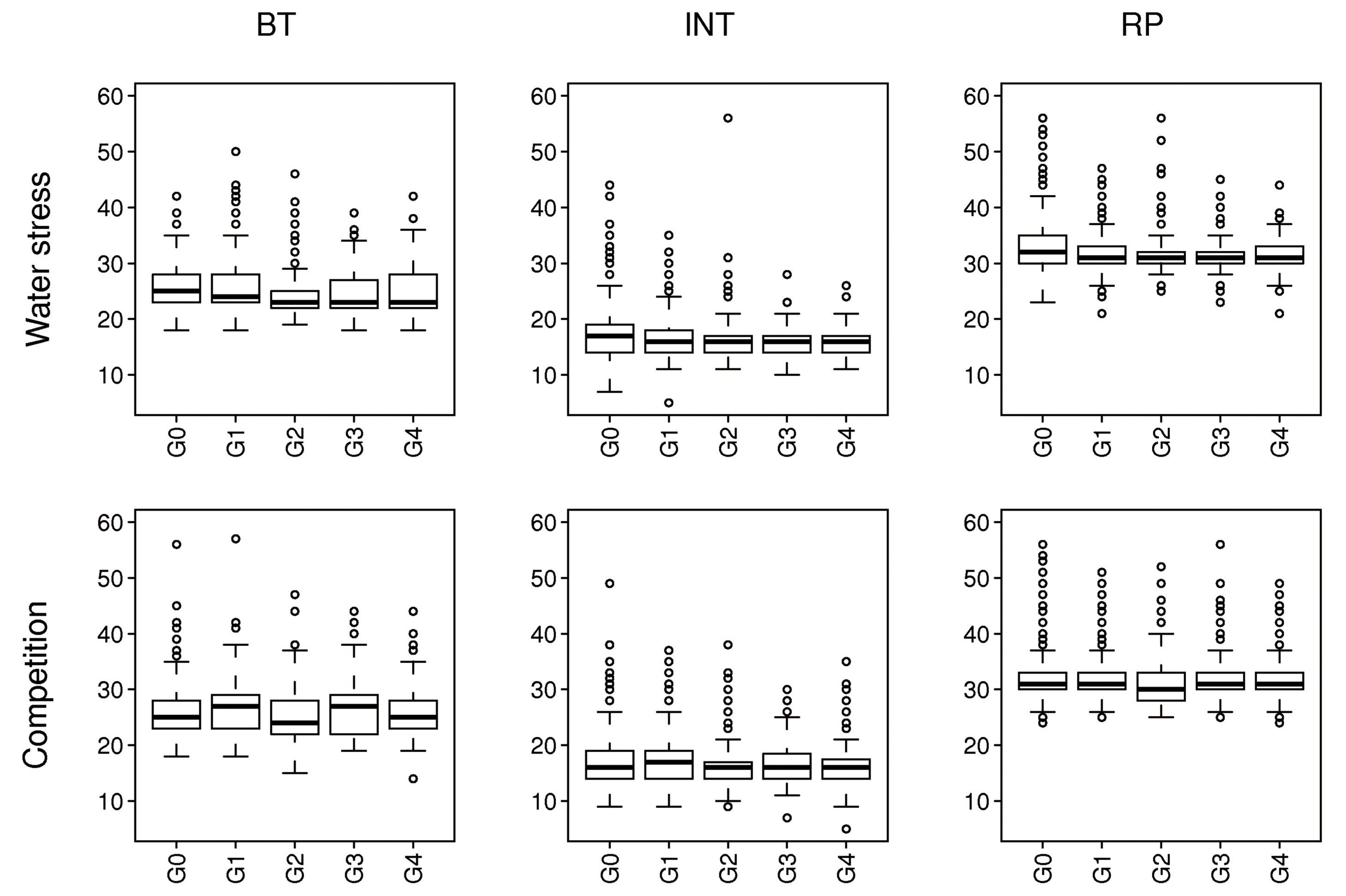

Supplement: Figure S2 — Evolution of phenological traits in experimental populations of Arabidopsis thaliana in the ‘water stress’ and ‘competition’ treatments. BT: bolting time, INT: interval between bolting and anthesis, RP: reproductive period duration. BT, INT and RP are expressed in days. G0: initial experimental generation, G1–G4: four successive experimental generations. For both treatments, raw data from the five intensities were pooled and are presented for each experimental generation. (TIFF) [file pone.0032069.s002.tif]

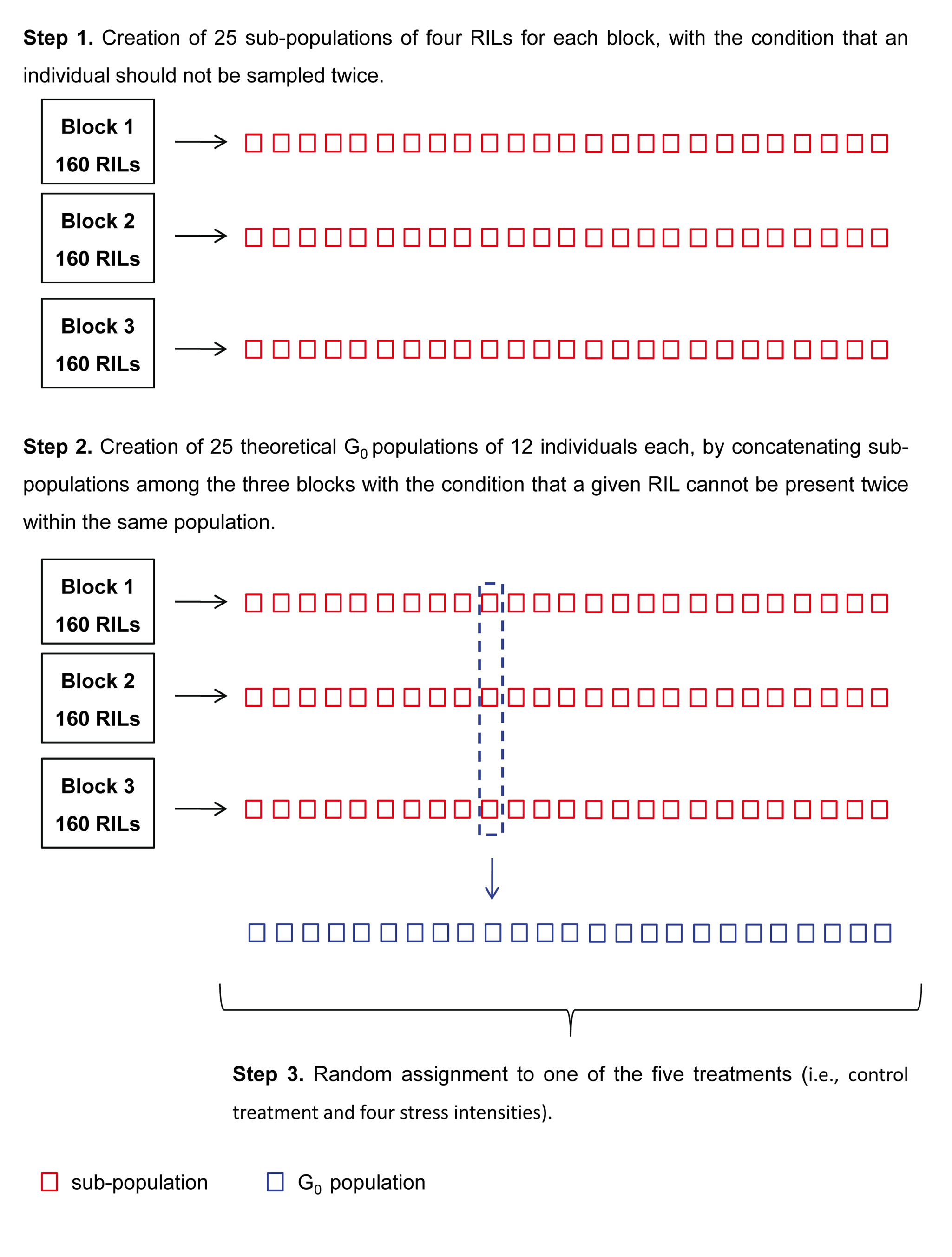

Supplement: Figure S3 — Theoretical populations for the initial generation G0 of the experiment set up for each type of environmental stress treatment (i.e., water stress and competition). (TIF) [file pone.0032069.s003.tif]
