# Supplementary material for: Adaptive Value of Phenological Traits in Stressful Environments: Predictions Based on Seed Production and Laboratory Natural Selection
Source: PLoS One. 2012 Mar 5;7(3):e32069. doi: 10.1371/journal.pone.0032069 (PMC3293886; doi:10.1371/journal.pone.0032069)
Supplement: Table S1 — Effects of treatment and genotype on phenological traits and fitness. (DOC) [file pone.0032069.s004.doc]

**Table S1.** Effects of treatment and genotype on phenological traits and fitness. GERM: germination timing, BT: bolting time, INT: interval between bolting and anthesis, ANT: anthesis, FLO: flowering, RP: reproductive period duration, FRR: flowering-to-reproductive period ratio, FITNESS: total silique length as a proxy of seed production.

| **Trait** | **Source^a^** | **d.f.** | **MS** | ***F*** | ***P*** |
| --- | --- | --- | --- | --- | --- |
| **GERM** | block | 10 | 0.21 | 22.30 | *** |
|  | genotype | 161 | 0.04 | 3.86 | *** |
|  | treatment | 4 | 0.02 | 2.33 | ns |
| **BT** | block | 10 | 2 x 10^-5^ | 1.69 | ns |
|  | genotype | 161 | 7.82 x 10^-5^ | 7.87 | *** |
|  | treatment | 4 | 2.5 x 10^-4^ | 1.28 | ns |
| **INT** | genotype | 161 | 1.16 | 2.16 | *** |
|  | treatment | 4 | 22.49 | 42.05 | *** |
| **ANT** | block | 10 | 1.3 x 10^-7^ | 2.47 | ** |
|  | genotype | 161 | 6.21 x 10^-7^ | 9.03 | *** |
|  | treatment | 4 | 2.72 x 10^-7^ | 5.35 | *** |
| **FLO** | genotype | 1 | 1.85 | 2.46 | *** |
|  | treatment | 161 | 56.34 | 74.99 | *** |
| **RP** | genotype | 161 | 2.6 x 10^-4^ | 1.63 | *** |
|  | treatment | 2 | 0.07 | 41.32 | *** |
| **FRR** | genotype | 161 | 0.15 | 1.3 | ** |
|  | treatment | 2 | 1.82 | 16.32 | *** |
| **FITNESS** | block | 10 | 316.4 | 3.80 | *** |
|  | genotype | 161 | 416.83 | 5.00 | *** |
|  | treatment | 4 | 9603.75 | 115.24 | *** |

The significance of the effects was tested after model selection based on Akaike’s information criterion (AIC). The ‘treatment x genotype’ and/or ‘block’ factors were eliminated from the complete model when these factors led to an increase of more than 2 points in the AIC (see Table S2). *0.05 > *P* > 0.01, **0.01 > *P* > 0.001, ****P* < 0.001, ns: non-significant

^a^ ‘block’ is nested in ‘treatment’.
