# Supplementary material for: Adaptive Value of Phenological Traits in Stressful Environments: Predictions Based on Seed Production and Laboratory Natural Selection
Source: PLoS One. 2012 Mar 5;7(3):e32069. doi: 10.1371/journal.pone.0032069 (PMC3293886; doi:10.1371/journal.pone.0032069)
Supplement: Table S2 — Model selection based on the Akaike's information criterion (AIC). (DOC) [file pone.0032069.s005.doc]

**Table S2. Model selection based on the Akaike’s information criterion (AIC). GERM: germination timing, BT: bolting time, INT: interval between bolting and anthesis, ANT: anthesis, FLO: flowering, RP: reproductive period duration, FRR: flowering-to-reproductive period ratio, FITNESS: total silique length as a proxy of seed production.**

|  | | |  | |  |  | |
| --- | --- | --- | --- | --- | --- | --- | --- |
| Trait | | | Model | |  | AIC | |
| GERM | | | Complete | | germ*_ijk_*= µ *_germ_* + block *_i_* (treatment *_j_*) + treatment *_j_* + genotype *_k_* + (treatment x genotype) *_jk_* + ε *_ijk_*. | -9965 | |
|  | | | Final | | germ*_ijk_*= µ *_germ_* + block *_i_* (treatment *_j_*) + treatment *_j_* + genotype *_k_* + ε *_ijk_*. | -10584 | |
| BT | | | Complete | | bt*_ijk_*= µ *_bt_* + block *_i_* (treatment *_j_*) + treatment *_j_* + genotype *_k_* + (treatment x genotype) *_jk_* + ε *_ijk_*. | -25710 | |
|  | | | Final | | bt*_ijk_*= µ *_bt_* + block *_i_* (treatment *_j_*) + treatment *_j_* + genotype *_k_* + ε *_ijk_*. | -26462 | |
| INT | | | Complete | | int*_ijk_*= µ *_int_* + block *_i_* (treatment *_j_*) + treatment *_j_* + genotype *_k_* + (treatment x genotype) *_jk_* + ε *_ijk_*. | -766 | |
|  | | | Final | | int*_ijk_*= µ *_int_* + treatment *_i_* + genotype *_j_* + ε *_ij_*. | -1286 | |
| ANT | | | Complete | | ft*_ijk_*= µ *_ft_* + block *_i_* (treatment *_j_*) + treatment *_j_* + genotype *_k_* + (treatment x genotype) *_jk_* + ε *_ijk_*. | | -37805 |
|  | | | Final | | ft*_ijk_*= µ *_ft_* + block *_i_* (treatment *_j_*) + treatment *_j_* + genotype *_k_* + ε *_ijk_*. | -38594 | |
| FLO | | | Complete | | fp*_ijk_*= µ *_fp_* + block *_i_* (treatment *_j_*) + treatment *_j_* + genotype *_k_* + (treatment x genotype) *_jk_* + ε *_ijk_*. | -72 | |
|  | | | Final | | fp*_ijk_*= µ *_fp_* + treatment *_i_* + genotype *_j_* + ε *_ij_*. | -496 | |
| RP | | | Complete | | rp*_ijk_*= µ *_rp_* + block *_i_* (treatment *_j_*) + treatment *_j_* + genotype *_k_* + (treatment x genotype) *_jk_* + ε *_ijk_*. | -8369 | |
|  | | | Final | | rp*_ijk_*= µ *_rp_* + treatment *_i_* + genotype *_j_* + ε *_ij_*. | -8581 | |
| FRR | | | Complete | | frr*_ijk_*= µ _fr_*_r_* + block *_i_* (treatment *_j_*) + treatment *_j_* + genotype *_k_* + (treatment x genotype) *_jk_* + ε *_ijk_*. | -2544 | |
|  | | | Final | | frr*_ijk_*= µ *_frr_* + treatment *_i_* + genotype *_j_* + ε *_ij_*. | -2848 | |
| FITNESS |  |  | | Complete | fitness*_ijk_*= µ *_fitness_* + block *_i_* (treatment *_j_*) + treatment *_j_* + genotype *_k_* + (treatment x genotype) *_jk_* + ε *_ijk_*. | 10626 | |
|  |  |  | | Final | fitness*_ijk_*= µ *_fitness_* + block *_i_* (treatment *_j_*) + treatment *_j_* + genotype *_k_* + ε *_ijk_*. | 10165 | |
