# Supplementary material for: Adaptive Value of Phenological Traits in Stressful Environments: Predictions Based on Seed Production and Laboratory Natural Selection
Source: PLoS One. 2012 Mar 5;7(3):e32069. doi: 10.1371/journal.pone.0032069 (PMC3293886; doi:10.1371/journal.pone.0032069)
Supplement: Table S4 — Genotypic selection analysis: comparison of selection gradients (β) and quadratic selection coefficients (γ) for phenological traits between the two intensities in each stress treatment (‘water stress’ and ‘competition’). (DOC) [file pone.0032069.s007.doc]

**Table S4. Genotypic selection analysis: comparison of selection gradients (β) and quadratic selection coefficients (γ) for phenological traits between the two intensities in each stress treatment (‘water stress’ and ‘competition’). GERM: germination timing, BT: bolting time, INT: interval between bolting and anthesis, FLO: flowering, RP: reproductive period duration, FRR: flowering-to-reproductive period ratio.**

| **Trait** |  | **Water stress (moderate *vs*. severe)** | | **Competition (moderate *vs*. intense)** | |
| --- | --- | --- | --- | --- | --- |
|  |  | *F* | *P* | *F* | *P* |
| GERM | β | **3.88** | ***** | 1.35 | ns |
|  | γ | 1.21 | ns | 0.08 | ns |
| BT | β | **75.94** | ******* | 0.47 | ns |
|  | γ | **6.66** | ***** | 1.58 | ns |
| INT | β | **18.69** | ******* | 0.79 | ns |
|  | γ | 0.42 | ns | 1.2 | ns |
| FLO | β | **7.02** | ******* | 1.87 | ns |
|  | γ | 0.01 | ns | 5.5 | ns |
| RP | β | NE | NE | 2.15 | ns |
|  | γ | NE | NE | 0.92 | ns |
| FRR | β | NE | NE | 2.45 | ns |
|  | γ | NE | NE | 4.62 | ns |

NE: not estimated. *0.05 > *P* > 0.01, **0.01 > *P* > 0.001, ****P* < 0.001, ns: non-significant.
